# Tropical rainfall over the last two millennia: evidence for a low-latitude hydrologic seesaw

**DOI:** 10.1038/srep45809

**Published:** 2017-04-05

**Authors:** Franziska A. Lechleitner, Sebastian F. M. Breitenbach, Kira Rehfeld, Harriet E. Ridley, Yemane Asmerom, Keith M. Prufer, Norbert Marwan, Bedartha Goswami, Douglas J. Kennett, Valorie V. Aquino, Victor Polyak, Gerald H. Haug, Timothy I. Eglinton, James U. L. Baldini

**Affiliations:** 1 Geological Institute, Swiss Federal Institute of Technology Zurich (ETHZ), Sonneggstrasse 5, CH-8092 Zurich, Switzerland; 2 Department of Earth Sciences, University of Durham, Durham, DH1 3LE, UK; 3 Sediment- and Isotope Geology, Institute for Geology, Mineralogy & Geophysics, Ruhr-Universität Bochum, Universitätsstr. 150, 44801 Bochum, Germany; 4 Alfred-Wegener-Institut Helmholtz-Zentrum für Polar- und Meeresforschung, Telegrafenberg A43, 14471 Potsdam, Germany; 5 Department of Earth and Planetary Sciences, University of New Mexico, Albuquerque, New Mexico, 87131 USA; 6 Department of Anthropology, University of New Mexico, Albuquerque, New Mexico, 87131 USA; 7 Potsdam Institute for Climate Impact Research, P.O. Box 60 12 03, 14412 Potsdam, Germany; 8 Department of Physics, Universität Potsdam, Karl-Liebknecht-Str. 24-25, 14476 Potsdam, Germany; 9 Department of Anthropology and Institutes for Energy and the Environment, The Pennsylvania State University, University Park, PA 16802, USA; 10 Department of Climate Geochemistry, Max Planck Institute for Chemistry, 55128 Mainz, Germany

## Abstract

The presence of a low- to mid-latitude interhemispheric hydrologic seesaw is apparent over orbital and glacial-interglacial timescales, but its existence over the most recent past remains unclear. Here we investigate, based on climate proxy reconstructions from both hemispheres, the inter-hemispherical phasing of the Intertropical Convergence Zone (ITCZ) and the low- to mid-latitude teleconnections in the Northern Hemisphere over the past 2000 years. A clear feature is a persistent southward shift of the ITCZ during the Little Ice Age until the beginning of the 19th Century. Strong covariation between our new composite ITCZ-stack and North Atlantic Oscillation (NAO) records reveals a tight coupling between these two synoptic weather and climate phenomena over decadal-to-centennial timescales. This relationship becomes most apparent when comparing two precisely dated, high-resolution paleorainfall records from Belize and Scotland, indicating that the low- to mid-latitude teleconnection was also active over annual-decadal timescales. It is likely a combination of external forcing, i.e., solar and volcanic, and internal feedbacks, that drives the synchronous ITCZ and NAO shifts via energy flux perturbations in the tropics.

Hemispheric antiphasing of large-scale precipitation patterns in low- and mid-latitude regions, driven by the seasonal migration of the Intertropical Convergence Zone (ITCZ), has been described over orbital timescales, Dansgaard-Oeschger (DO), and Heinrich events^1^,^2^,^3^. As part of the upward limb of the Hadley Cells, the ITCZ plays a crucial role in global energy redistribution. Temperature-driven meridional displacement of the tropical Hadley Cells and the ITCZ^4^,^5^ induced synchronous shifts in higher latitude climate patterns^1^ on millennial timescales. High signal-to-noise ratios of millennial-scale climate shifts during glacial periods, largely due to different boundary conditions related to the presence of extensive continental ice sheets, facilitate their detection in proxy records.

Although this atmospheric reorganization has been described over glacial-interglacial timescales, the dynamics and latitudinal extent of this interhemispheric hydrologic seesaw^1^ over the most recent past are still poorly understood. Additionally, chronological uncertainties, although less significant than in Pleistocene reconstructions, and low signal-to-noise ratios in Holocene paleoclimate records can hinder interpretations of rapid climate change, and this is particularly true over the last few millennia.

Here we investigate the latitudinal extent of the hydrologic seesaw in the Northern Hemisphere (NH) over the past two millennia by comparing precisely dated, high-resolution paleo-rainfall records from low- and mid-latitudes. We reconstruct broad ITCZ migrations using 25 published high-resolution stalagmite, sediment, tree ring, and ice core records from both hemispheres identified as reflecting low-latitude, ITCZ-driven rainfall, before extending the comparison to NH mid-latitudes. Details of the incorporated records are presented in Suppl. Table 1. Because our reconstruction focuses on the total extent of hemispheric displacement of the ITCZ, only records experiencing one annual passage of the ITCZ or with a clear bias towards one rainy season are considered. Records were selected based on sampling resolution (<15 years on average), chronological precision (mean 2σ error < 40 years), and location, in order to maximize spatial coverage and interpretative value (Fig. 1). To reconstruct long-term ITCZ migration dynamics, the records were combined into hemispheric stacks relative to their location (Fig. 2), and then to an overall ITCZ-stack (Fig. 3), by bringing them onto a common timescale and averaging their signal (normalized as z-scores, see Methods). To confirm that no bias was introduced with our selection of records, a second stack was compiled, which included records that did not strictly meet the selection criteria (due to low resolution and/or insufficiently precise dating). Comparison between the two stacks shows very little difference, indicating that our selection of records captures trends at the global scale (Suppl. Figure 1).

Stacking the records for each hemisphere results in positive z-scores indicating drier conditions, and negative z-scores indicating wetter conditions (Fig. 2). Due to chronological uncertainties, we consider only decadal-centennial scale trends within these stacks. Both hemispheric stacks show relatively stable conditions between 0 to ~1320 C.E., with the exception of a dry interval evident in some NH records during the 11^th^ Century (Fig. 2A). This 11th Century excursion is not observed in SH records (Fig. 2B). The most prominent, and hemispherically antiphased, shift in both records occurred between 1320–1820 C.E., indicating a pronounced southward displacement of the ITCZ during this period (Fig. 2C). These longer-term features become even more apparent when the two stacks are combined into one ITCZ-stack record, reflecting the deflection of the ITCZ over the past 2000 years (positive – ITCZ is positioned more northwards, negative – ITCZ is positioned more southwards). In this combined record the most pronounced period of southward deflection of the ITCZ also occurred between 1320 and 1820 C.E. (Fig. 3).

We then investigated the long-term (decadal-centennial scale) latitudinal extent of this hydrologic seesaw between tropical regions and the mid-latitude North Atlantic by comparing the ITCZ-stack to a recent 1000-year-long, model-constrained North Atlantic Oscillation (NAO_mc_) reconstruction based on records spanning broad regions of the western NH^6^ (Fig. 3). NAO_mc_ also includes datasets from high latitudes (i.e., northern Canada, Greenland, northern Scandinavia), but because the NAO is a leading pattern of weather and climate variability in the NH mid-latitudes^7^ it is indicative of mid-latitude hydroclimate conditions. Comparison of decadal-centennial trends in both reconstructions reveals compelling similarities (r = 0.78, p < 0.001, calculated using standard correlation and a prior transformation of the time series to normal distribution, Fig. 3), implying that ITCZ migration and NAO variability were closely coupled over the last 1,000, and most likely the last 2,000, years.

Two very precisely dated, high-resolution records constrain higher frequency hydroclimate variability from the low- and mid-latitudes, since small-scale variability is averaged out in the ITCZ stacks (Fig. 4, Suppl. Figure 2). We use stable carbon isotope ratios (δ^13C^) from stalagmite YOK-I from Yok Balum Cave in southern Belize^8^ as the low-latitude end-member, because it is one the highest resolved (0.5 years on average) and best dated (mean error 3.3 years) records from the ITCZ-stack that covers the entire period of the reconstruction (Suppl. Table 1). Variations in δ^13^C at this cave site have previously been attributed to variations in rainfall registered above the cave, governed by the seasonal migration of the ITCZ^8^,^9^. Therefore, δ^13^C at Yok Balum Cave is a more sensitive proxy to variations in regional rainfall amount (and particularly drying) than δ^18^O, which is a mixed signal including precipitation amount, moisture source, and storm path length^9^,^10^. Yok Balum Cave is located at the northernmost extent of the boreal summer ITCZ, a remarkably sensitive location to record even small shifts in ITCZ position^9^. We compare the Belizean ITCZ record to the well-dated growth-band width record from stalagmite SU-96-7 from Uamh-an-Tartair Cave in Scotland^11^. The SU-96-7 record is highly correlated to precipitation in Scotland, and consequently to the winter NAO^11^,^12^, and was also included in the NAO_mc_ composite. This record was preferred over a more recent composite NAO reconstruction from Scotland^12^ (which includes SU-96-7), because of its higher sensitivity to the NAO (r = −0.70^11^ vs. r = −0.46^12^), which may reflect different hydrological pathways feeding the individual stalagmites in the composite records, thus smoothing the NAO signal. This would likely remove high frequency trends, leading to the loss of key events at the (sub)decadal scale.

The long-term trend in both records was identified and removed by using a Savitzky-Golay smoothing filter, thus highlighting subdecadal-scale variations and facilitating comparison (Suppl. Figure 2). Several shared multi-annual drying events are clearly present within both residual time series (r = 0.51, p < 0.001) (Fig. 4, Suppl. Figure 3). Particularly notable is the almost century-long event recorded at both locations between ~1020–1100 C.E., one of a series of multi-decadal droughts that contributed to the transformation of Classic Maya society in Central America^8^. The similarity in both the smoothed long-term trends and sub-decadal variations between the low-latitude YOK-I record and the mid-latitude SU-96-7 record suggests that teleconnections exist between these two locations, linked through the Bermuda-Azores High.

Paleoclimate records covering the last glacial period have advanced the concept of the interhemispheric hydrologic seesaw on millennial timescales; for example, marine records from the Cariaco Basin and the Arabian Sea show pronounced long-term southward displacement of the ITCZ during Heinrich stadials^3^. At the same time, stalagmites from China record reduced summer precipitation and a weaker Asian Summer Monsoon (ASM)^13^,^14^. During Greenland warming episodes, stalagmite records from Peru and Brazil show that this coincided with periods of decreased South American Summer Monsoon (SASM) strength^2^,^15^. Our stacked ITCZ record reveals that hemispheric antiphasing of low latitude precipitation over the last 2000 years is due to meridional displacement of the ITCZ and related low-latitude climate patterns, rather than of overall weakened precipitation in the tropics^4^,^16^. The hydrologic seesaw results from perturbations in tropical energy flux, e.g., due to hemispheric temperature contrasts, as the ITCZ tracks the thermal equator^17^,^18^. Meridional displacement of the ITCZ triggers shifting of the Hadley cells in both hemispheres^17^, and consequently drives circulation processes at higher latitudes. A long-term global cooling trend^19^,^20^ coincident with a pronounced southward shift of the ITCZ from ~1300 C.E. onwards support this hypothesis (Fig. 3). Globally cooler temperatures would provoke a weakening of the Atlantic meridional overturning circulation (AMOC) and increased Arctic sea ice, resulting in a southward shift of the energy flux equator and the ITCZ^17^.

The Yok Balum, Forestry, and Dante Cave records are directly influenced by the mean position of the ITCZ^8^,^21^,^22^,^23^ (with the Forestry Cave record being influenced primarily by the South Pacific Convergence Zone, the most persistent spur of the ITCZ^23^,^24^). Monsoonal systems, like the ASM, the SASM, the Australian-Indonesian Summer Monsoon (AISM), the Indian Summer Monsoon (ISM), and the West African Monsoon (WAM) are affected by the hemispheric migration of the ITCZ as a major moisture flux conduit and by hemispheric temperature, because land-sea temperature contrasts drive the monsoonal systems^17^,^25^. Although monsoonal variations might not necessarily be equivalent to ITCZ shifts, monsoons can be linked to ITCZ variations^17^,^26^,^27^,^28^. The SASM (Huagapo, Cascayunga, Curupira, and Pau d’Alho Caves, and Quelccaya Ice cap) and the AISM (Chillagoe Cave, KNI-51 Cave) records used here are all antiphased with respect to the NH records on decadal-centennial scales, indicating stronger SH monsoons when NH low latitudes are drier. The similarities between these globally distributed records, and the consistent hemispheric antiphasing over decadal-centennial timescales, are compelling and suggest that the interhemispheric hydrologic seesaw was active, albeit in subdued fashion compared to the Last Glacial, over the most recent past. However, additional precisely dated high-resolution records from the tropics are necessary to gain more detailed insights into possible different regional expressions of hydroclimate variability.

Propagation of meridional ITCZ shifts to mid-latitude regions in both hemispheres occurred across millennial-scale climate shifts: for example, a stalagmite δ^18^O record from New Mexico, U.S., shows increased winter precipitation during NH cooling phases^5^, and stalagmite growth frequencies in Korea and south-eastern Australia are inversely correlated^1^. Southward shifts of the ITCZ and the Hadley Cells during cold phases reduce the meridional pressure gradient in the NH, inducing expansion and southward displacement of the polar and mid-latitude pressure cells. Consequently, the polar jet and westerlies shifted southward and weakened^5^, and monsoonal systems propagated less far northward^1^. At the same time, southward displacement and compression of the SH pressure cells results in strengthening and southward displaced mid- and high-latitude wind and weather patterns^1^,^4^,^29^. The significant correlation between our ITCZ-stack and the NAO_mc_ record indicates that these mechanisms very likely existed during the last millennium, as well as during the Last Glacial. Colder NH temperatures and a southward-shifted ITCZ would promote negative NAO conditions, due to a lower meridional pressure gradient, as well as weakening and southward shift of the NH westerlies, whereas a northward displacement of the ITCZ may trigger a positive NAO^30^ (Fig. 3). However, we cannot at present rule out the potential influence of additional atmospheric mechanisms, e.g., related to changes in moisture transport and convection activity along the ITCZ, as well as in the Walker circulation and the El-Niño Southern Oscillation (ENSO), which could impact the distribution of low-latitude rainfall patterns. Similarly, conditions in the North Atlantic related to sea ice extent, temperature, and snow cover, could lead to simultaneous, but unrelated changes in both the NAO and the ITCZ, possibly with influences from the AMOC. In particular, the expression of these mechanisms will greatly vary on the regional scale, and it is also possible that both realms react to a certain extent independently to the common forcing from hemispheric or global temperature gradients.

The two millennia preceding industrialization were characterized by a long-term cooling trend, culminating in a well-documented period of globally colder conditions, the Little Ice Age (LIA, ~1200–1850 C.E.)^19^,^20^,^31^. The presence of a globally synchronous warm period between ~950–1200 C.E.^32^, the Medieval Climate Anomaly (MCA), is being challenged by more recent global temperature reconstructions^19^,^20^, and is also not expressed as a change in the latitudinal position of the ITCZ in our stacked ITCZ records (Fig. 3). However, it is noteworthy that three out of the six NH records included in the stack that cover the MCA interval indicate drier conditions between 1000–1100 C.E. (Fig. 2), and both high-resolution records considered here, YOK-I and SU-96-7, show simultaneous and persistent drying occurring in Scotland and Belize between 1020–1100 C.E. (Fig. 4). These records support tree ring reconstructions from Europe and North America^33^,^34^, and lake sediment reconstructions from equatorial Africa^35^, all reflecting regional NH drought during the 11th century C.E. At the same time, wetter conditions were registered in a stalagmite record from Madagascar (15°S)^36^. It is possible that this resulted from reduced solar irradiance during the contemporaneous Oort solar minimum (centered around 1050 C.E.).

The low-latitude hydroclimate records discussed here all suggest a southward ITCZ shift broadly synchronous with the LIA (here 1320–1820 C.E.), and that the ITCZ only began migrating northwards again after ~1820 C.E., with the beginning of the Current Warm Period (CWP) (Fig. 3). The persistence of LIA cooling has been documented before and appears to be globally expressed^19^, consistent with the concept of an energy flux perturbation in the tropics and associated southward displacement of the ITCZ due to weakening of the AMOC (and increased ice cover in the Arctic Ocean)^17^ (Fig. 3). The causes for the extensive LIA cooling in the NH remain enigmatic, but volcanic forcing appears to have been dominant during this time^19^,^31^,^37^. Stratospheric sulfate aerosols from explosive volcanic eruptions affect climate on annual to decadal timescales via scattering and absorption of solar radiation^38^,^39^,^40^. Large NH volcanic eruptions have been linked to hemispheric displacement of the ITCZ and related circulation patterns by cooling the hemisphere of the eruption, resulting in hemispheric temperature asymmetries^9^,^16^,^18^,^29^. Several large volcanic eruptions occurred during the late 13th century most notably the 1257 C.E. Samalas/Rinjani eruption^41^, with an estimated sulfate load of 73 kg km^−2^ 42.

Protracted global cooling may have resulted from the amplifying effects of expanding sea ice and snow cover in northern latitudes^31^,^43^.

Changes in solar activity are another proposed cause for the widespread NH cooling during the LIA^44^,^45^,^46^,^47^. A cluster of four significant “grand solar minima” occurred within the LIA, whereas solar activity during previous centuries was higher. It is possible that the combination of low solar activity and NH volcanic eruptions with associated feedbacks (such as from increased sea ice and more frequent atmospheric blocking events over the North Atlantic)^31^,^47^ between 1250–1800 C.E^31^,^47^, led to the LIA cooling and southward ITCZ displacement. The importance of insolation changes is however much smaller than that of volcanic eruptions in terms of radiative forcing. It is therefore likely that the volcanic forcing dominated, while changes in solar activity enhanced these trends.

It is worth noting that some of the short-lived events in the Scottish and Belizean high-resolution records appear linked with the volcanic record: the clearest connection appears between the 1783 C.E. *Laki*/*Grimsvötn* eruption, the largest NH eruption of the last 1000 years in terms of sulfate loads^40^, occurring synchronously with strong drying in Belize and reduced rainfall in Scotland (Fig. 4). The 1783 C.E. event was previously also described in the YOK-G stalagmite record from Yok Balum Cave and coincides with the peak of the strongest pre-industrial drought since 1550 C.E. in Belize^9^. Additionally, we find indication of a response to volcanic forcing for the 934 C.E. *Eldgja* eruption in YOK-I, as well as for the 1458 C.E. *Kuwae* eruption in SU-96-7. Different responses between YOK-I and SU-96-7 could be related to the season of the eruption (rainfall is highest during winter in Scotland, and during summer in Belize), or to differing aerosol transport paths having very different latitudinal climate impacts. Moreover, chronological uncertainties in both the YOK-I and SU-96-7 records do not allow for a definitive attribution of these events to a volcanic trigger, and remain tentative.

We note that most of the short-lived events recorded in Belize lag the events in Scotland by ~15–20 years. This relationship is corroborated by the standard correlation, which is maximized at a lag of 16 years, and by a cross correlation analysis performed for the two time series (Fig. 4), which shows that nearly all the visually identified events are also characterized by significant positive correlations with lags of up to 40 years (with SU-96-7 leading). It is possible that such a lagged response of precipitation to solar forcing is more rapidly translated to the North Atlantic than to the Caribbean region (e.g., via sea ice feedbacks). However, we note that the lag we find is too close to the chronological uncertainty in the two time series to be robustly assigned to climatic phenomena.

Our results suggest that the low- to mid-latitude hydrologic seesaw is a feature inherent to the climate system at very different timescales, with only the strength of its expression varying. The presence, extent and dynamics of the hydrologic seesaw over the past two millennia is remarkable given the very different boundary conditions of the global climate system compared to glaciations, indicating that more subtle variations in the hemispheric temperature gradient are sufficient to change the meridional position of the ITCZ and the subtropical highs. This observation is only possible because of the recent development of high quality records covering the last two millennia. We suggest that, as chronological precision is further improved and additional records become available, the hydrologic seesaw may become resolvable at (multi-)annual timescales as well.

## Methods

### Construction of the hemispheric and ITCZ stacks

Using the age modeling software COPRA^48^, ensembles of 2,000 realizations of each record’s age model were computed. Subsequently, a mild tuning was performed to find the best age model within the ensemble of each individual record, defined as the age model that maximizes the signal correlation against all other overlapping records. A 2,000 year nonlinear (Gaussian) trend was subtracted from the records prior to correlation estimation to focus the alignment on centennial timescales and improve the signal-to-noise ratio. Correlations between records used in the stack are estimated directly from the irregular time series using Gaussian kernel correlation^49^. The best realizations for each proxy record are then brought to a common resolution of 10 years in a double-interpolation routine that minimizes aliasing of high-frequency variability into the result^50^. The stacks are given by the unweighted average of the standardized and centralized records. An estimate of the uncertainty of this average is gained from the standard error of the mean, taking into account the number of records averaged at each point. This error represents a lower bound for the true uncertainty, as it implicitly assumes independence amongst all datasets. The assumption of independence may further give rise to a regional bias or site bias, considering that the records are not distributed equally spaced around the Earth and some are closely spaced. A detailed inspection of the potential impact of this location bias, for example by pseudoproxy experiments using climate model output would require knowledge of (or further assumptions about) the proxy signal, the signal-to-noise ratio and chronological uncertainties. Hence, such a comparison is currently beyond the scope of this manuscript.

### Cross-correlation analysis between YOK-I and SU-96-7

To estimate the cross-correlation at various lags for the YOK-I δ^13^C and SU-96-7 band width records, we use the framework of kernel-based cross-correlation analysis by Rehfeld and Kurths (2014)^49^ implemented in the toolbox NESTool (http://tocsy.pik-potsdam.de/nest.php). We move a window of 100 years from ~900 C.E. to ~2000 C.E. and after extracting the portion of the record within a window, we estimate the correlation with the YOK-I record being lagged up to 60 years. Statistical significance of the correlation values is determined with 1000 randomised surrogates of the datasets at a confidence level of alpha = 0.05, corrected for multiple comparisons using the Bonferroni correction factor.

## Additional Information

**How to cite this article:** Lechleitner, F. A. *et al*. Tropical rainfall over the last two millennia: evidence for a low-latitude hydrologic seesaw. *Sci. Rep.* **7**, 45809; doi: 10.1038/srep45809 (2017).

**Publisher's note:** Springer Nature remains neutral with regard to jurisdictional claims in published maps and institutional affiliations.

## Supplementary Material

Supplementary Material

## Figures and Tables

**Figure 1:**
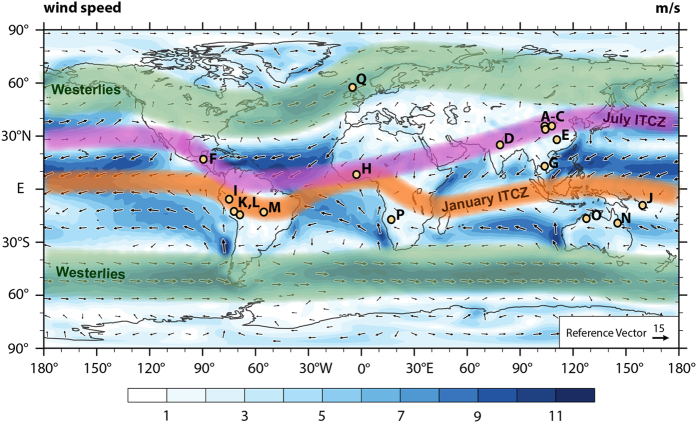
Map depicting the locations of the records presented in this study. A – Stalagmites HY1 and HY2 from Huangye cave, China^51^; B – Stalagmite WX42B from Wanxiang cave, China^52^; C – Stalagmite DY1 from Dayu Cave, China^53^; D – Stalagmites SAH-A and SAH-B from Sahiya Cave, India^54^; E – Stalagmite A1 from Lianhua Cave, China^55^; F – Stalagmite YOK-I from Yok Balum cave, Belize^8^; G – Tree ring reconstruction from Bidoup Nui Ba National Park, Vietnam^56^; H – Sediment record from Bosumtwi Lake, Ghana^57^; I – Stalagmite CAS-D from Cascayunga cave, Peru^58^; J – Stalagmites 10FC-02 and 05FC-04 from Forestry Cave, Guadalcanal, Solomon Islands^23^; K – Stalagmites P00-H1 and P09-H2 from Huagapo cave, Peru^59^; L – Ice core from the Quelccaya ice cap, Peru^60^; M – Stalagmites from Curupira and Pau d’Alho Caves, Brazil^61^; N – Stalagmite CH-1 from Chillagoe, Australia^62^; O – Stalagmites KNI-51 F, G, I, O, P, and 11 from KNI-51 Cave, Australia^63^; P –Stalagmite DP1 from Dante cave, Namibia^21^,^22^; Q – Stalagmite SU-96-7 from Uamh-an-Tartair cave, Scotland^11^. Winter wind vectors in the background are derived from 1950–2000 reanalysis data provided by the 20^th^ Century Reanalysis Composites from the NOAA Earth System Research Laboratory, Physical Science Division. The map was created using the NCAR Command Language (Version 6.3.0), 2016, Boulder, Colorado: UCAR/NCAR/CISL/TDD. http://dx.doi.org/10.5065/D6WD3XH5.

**Figure 2:**
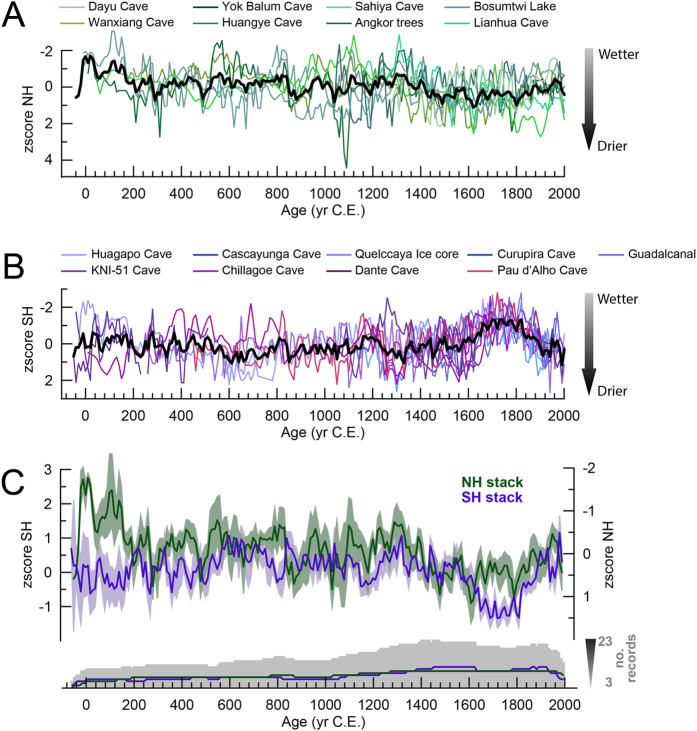
NH and SH ITCZ record stacks. All records have been converted to z-scores. (**A**) NH record compilation, the resulting stack is shown by the black line. (**B**) SH record compilation, the resulting stack is shown by the black line. (**C**) Both NH and SH stacks are shown with their uncertainties: antiphasing of the two stacks on centennial timescale becomes apparent, especially during the period 1320–1820 C.E.

**Figure 3:**
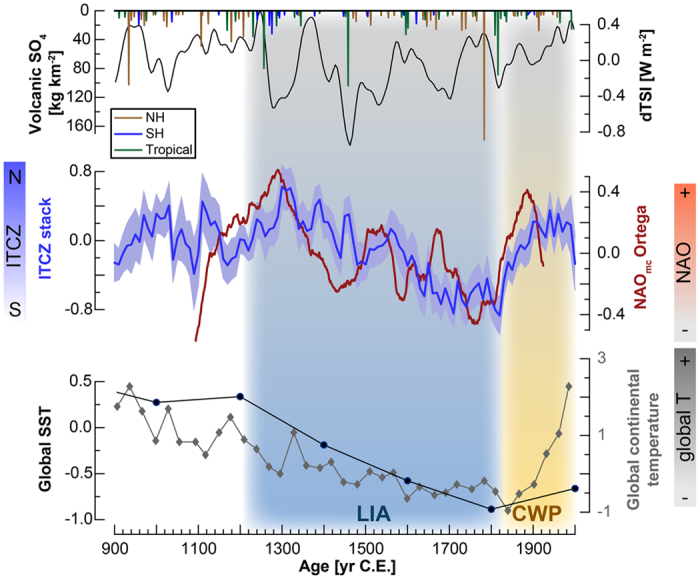
Comparison of records to the ITCZ-stack over the last ~1000 years. From top: Volcanic sulfate (SO_4_) recorded in ice cores from Antarctica and Greenland^40^, and solar forcing (dTSI) from ^10^Be in ice cores^64^. Note that solar and volcanic activity are plotted independently from their radiative forcing, which is much stronger for volcanic eruptions than for solar activity. Model-constrained multi-proxy NAO reconstruction by Ortega *et al*.^6^ with 91-point running average to highlight decadal-centennial trends. ITCZ-stack (this study), showing relative meridional ITCZ deflection over time. Global continental temperature reconstruction by the PAGES 2k consortium^19^ (grey line), and global sea surface temperature (SST) reconstruction by McGregor *et al*.^20^ (black line). The intervals of the Little Ice Age (LIA, ~1200–1850 C.E.) and Current Warm Period (CWP, after 1850), defined as in refs. 19, 20, 31 are highlighted with background shading.

**Figure 4:**
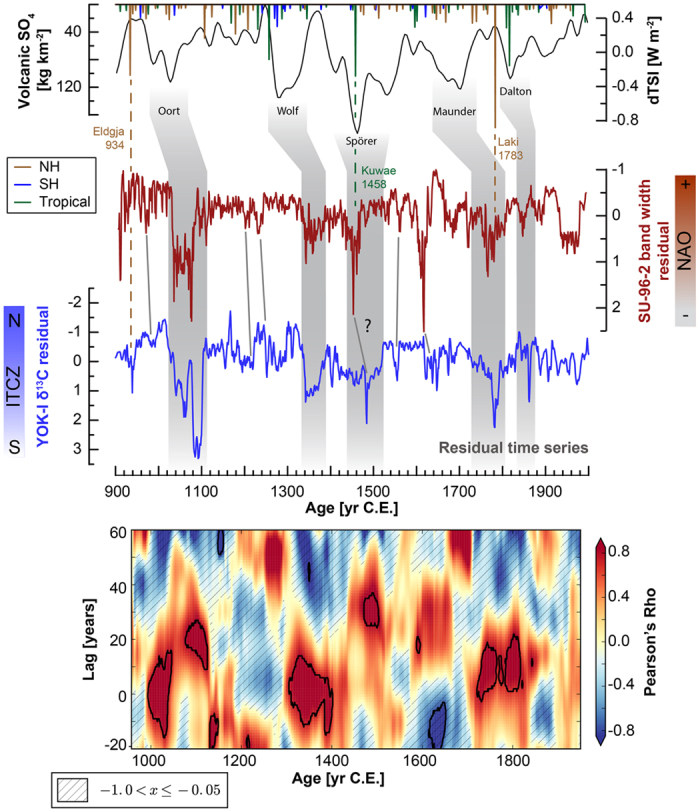
Comparison of short-term variations in hydroclimate between low- and mid-latitudes in the NH. The residuals of the smoothed YOK-I and SU-96-7 records are shown in the middle of the figure. Events thought to have occurred in both records are highlighted by grey lines. Volcanic sulfate recorded in ice cores from Antarctica and Greenland^40^, as well as solar forcing from^10^Be in ice cores^64^, are shown at the top of the figure. Volcanic eruptions tentatively identified in the proxy records are indicated by dashed lines and the eruption name and year. Solar minima recorded with a lag in the proxy records are shown by the grey bars. The color coded plot at the bottom of the figure shows the lagged cross correlation between the YOK-I δ^13^C and SU-96-7 band width records for their common time duration (~900–2000 C.E.). The time evolution of the lagged correlation was obtained using a sliding window of 100 years and allowing for a maximum lead of 60 years to the SU-96-7 record. We find statistically significant correlations between lags of 0–10 years at around 1000, 1300–1400, and 1700–1810 C.E., and lags of 10–20 years during the 1020–1100 C.E. event. Hatched regions in the plot indicate negative correlation values and statistically significant regions of correlation are marked with a thick black boundary.
