# Supplementary material for: Tropical rainfall over the last two millennia: evidence for a low-latitude hydrologic seesaw
Source: Sci Rep. 2017 Apr 5;7:45809. doi: 10.1038/srep45809 (PMC5381098; doi:10.1038/srep45809)
Supplement: Supplementary Material [file srep45809-s1.doc]

**Supplementary file for:**

**Tropical rainfall over the last two millennia: evidence for a low-latitude hydrologic seesaw**

Franziska A. Lechleitner^1,2^*, Sebastian F.M. Breitenbach^3^, Kira Rehfeld^4^, Harriet E. Ridley^2^, Yemane Asmerom^5^, Keith M. Prufer^6^, Norbert Marwan^7^, Bedartha Goswami^7,8^, Douglas J. Kennett^9^, Valorie V. Aquino^6^, Victor Polyak^5^, , Gerald H. Haug^1,10^, Timothy I. Eglinton^1^, James U.L. Baldini^2^

* To whom correspondence should be addressed. Email: franziska.lechleitner@erdw.ethz.ch

^1^ Geological Institute, Swiss Federal Institute of Technology Zurich (ETHZ), Sonneggstrasse 5, CH-8092 Zurich, Switzerland

^2^ Department of Earth Sciences, University of Durham, Durham, DH1 3LE, UK

**^3^** Sediment- and Isotope Geology, Institute for Geology, Mineralogy & Geophysics, Ruhr-Universität Bochum, Universitätsstr. 150, 44801 Bochum, Germany

^4^ Alfred-Wegener-Institut Helmholtz-Zentrum für Polar- und Meeresforschung, Telegrafenberg A43, 14471 Potsdam, Germany

^5^ Department of Earth and Planetary Sciences, University of New Mexico, Albuquerque, New Mexico, 87131 USA

^6^ Department of Anthropology, University of New Mexico, Albuquerque, New Mexico, 87131 USA

^7^ Potsdam Institute for Climate Impact Research, P.O. Box 60 12 03, 14412 Potsdam, Germany

^8^ Department of Physics, Universität Potsdam, Karl-Liebknecht-Str. 24-25, 14476 Potsdam, Germany

^9^ Department of Anthropology and Institutes for Energy and the Environment, The Pennsylvania State University, University Park, PA 16802, USA

^10^ Department of Climate Geochemistry, Max Planck Institute for Chemistry, 55128 Mainz, Germany

| **Record** | **Location** | **Coordinates** | **Archive** | **Proxy** | **Interval (in this study, yr C.E.)** | **Average 2s error** | **Resolution last 2000 years (yr)** | **Interpretation** | **Reference** |
| --- | --- | --- | --- | --- | --- | --- | --- | --- | --- |
| Huangye 1 | China | 33°35'N, 105°07'E | Stalagmite | δ^18^O | 760-1982 | 34.60 | 6.50 | ASM | 1 |
| Huangye 2 | China | 33°35'N, 105°07'E | Stalagmite | δ^18^O | 138-877 | 38.20 | 3.80 | ASM | 1 |
| *Huangye 3* | *China* | *33°35'N, 105°07'E* | *Stalagmite* | *δ^18^O* | *1308-2002* | *84.00* | *4.00* | *ASM* | 1 |
| Wanxiang | China | 33°19'N, 105°00'E | Stalagmite | δ^18^O | 192-2000 | 2.20 | 2.60 | ASM | 2 |
| Dayu | China | 33°08'N, 106°18'E | Stalagmite | δ^13^C | 1265-1982 | 2.40 | 1.33 | ASM | 3 |
| Sahiya | India | 30°36'N, 77°52'E | Stalagmite | δ^18^O | -140-2006 | 23.60 | 1.10 | ISM | 4 |
| Lianhua | China | 29°29'N, 109°32'E | Stalagmite | δ^13^C | 1140-2006 | 4.24 | 6.00 | ASM | 5 |
| *Dos Anas* | *Cuba* | *ca. 24°N, 85°W* | *Stalagmite* | *δ^18^O* | *691-2000* | *80.00* | *1.87* | *Hurricane rainfall* | 6 |
| *Dandak* | *India* | *19°00'N, 82°00'E* | *Stalagmite* | *δ^18^O* | *620-1578* | *61* | *1.30* | *ISM* | 7 |
| *Defore* | *Oman* | *17°07'N, 54°05'E* | *Stalagmite* | *δ^18^O* | *1230-1990* | *130* | *1.29* | *ISM* | 8 |
| Yok Balum | Belize | 16°12’N, W89°4’W | Stalagmite | δ^13^C | 0-2000 | 6.68 | 0.50 | ITCZ rainfall | 9 |
| Angkor | Cambodia | ca. 15°N, 105°E | Tree rings | PDSI | 1250-2008 | 2.00 | 1.00 | ASM | 10 |
| *Cariaco* | *Cariaco basin* | *10°42.73'N, 65°10.18'W* | *Ocean sediment* | *Ti %* | *-50-1650* | *131* | *4.44* | *ITCZ* | 11 |
| Bosumtwi | Ghana | 6°30'N, 1°25'W | Lake sediment | δ^18^O | -700-2003 | 2.00 | 4.60 | WAM | 12 |
| Cascayunga | Peru | 6°04'S, 77°11’W | Stalagmite | δ^18^O | 540-1900 | 12.00 | 1.75 | SASM | 13 |
| Forestry 5FC | Solomon Islands | 9°29'S, 159°58'E | Stalagmite | δ^18^O | 1880-1975 | 1.40 | 0.17 | SPCZ rainfall | 14 |
| Forestry 10FC | Solomon Islands | 9°29'S, 159°58'E | Stalagmite | δ^18^O | 1423-2010 | 1.72 | 0.27 | SPCZ rainfall | 14 |
| *Pumacocha* | *Peru* | *10°4’S, 76°3’W* | *Lake sediment* | *δ^18^O* | *0-2000* | *102.50* | *1.78* | *SASM* | 15 |
| Huagapo | Peru | 11°16’S, 75°35’W | Stalagmite | δ^18^O | 0-2000 | 8.20 | 3.67 | SASM | 16 |
| Quelccaya | Peru | 13°56'S, 70°50'W | Ice core | δ^18^O | 200-2000 | 2.00 | 1.00 | SASM | 17 |
| Curupira | Brasil | 15°12'S, 56°47'W | Stalagmite | δ^18^O | 1795-1970 | 33.16 | 0.70 | SASM | 18 |
| Pau d'Alho | Brasil | 15°12'S, 56°48'W | Stalagmite | δ^18^O | 490-1760 | 3.74 | 1.20 | SASM | 18 |
| KNI-51 F | Australia | 15.3°S, 128.6°E | Stalagmite | δ^18^O | 1085-1632 | 8.1 | 8 | AISM | 19 |
| KNI-51 G | Australia | 15.3°S, 128.6°E | Stalagmite | δ^18^O | 1310-1638 | 9.80 | 6.00 | AISM | 19 |
| KNI-51 I | Australia | 15.3°S, 128.6°E | Stalagmite | δ^18^O | -40-230 | 14.60 | 11.00 | AISM | 19 |
| KNI-51 O | Australia | 15.3°S, 128.6°E | Stalagmite | δ^18^O | 237-522 | 12.60 | 3.90 | AISM | 19 |
| KNI-51 P | Australia | 15.3°S, 128.6°E | Stalagmite | δ^18^O | -62-257 | 27.40 | 6.00 | AISM | 19 |
| KNI-51 11 | Australia | 15.3°S, 128.6°E | Stalagmite | δ^18^O | 1870-2008 | 6.60 | 4.50 | AISM | 19 |
| Chillagoe | Australia | ca. 17°S, 144°E | Stalagmite | δ^18^O | 1227-2003 |  | 1.00 | AISM | 20 |
| Dante | Namibia | 19°24'S, 17°53'E | Stalagmite | δ^18^O | 0-1939 | 37.80 | 10.70 | ITCZ rainfall | 21 |
| Dante_new | Namibia | 19°24'S, 17°53'E | Stalagmite | δ^18^O | 1463-1939 | 21.20 | 1.81 | ITCZ rainfall | 22 |

Suppl. Table 1: Summary of all used proxy records for the ITCZ reconstruction. Records in italics did not meet the selection criteria applied for the stack calculation, and were only used for control low-resolution calculations.

Suppl. Fig. 1: Comparison of the main stack, including only records that meet the selection criteria, with a calculated stack including also the low resolution records from Suppl. table 1 (in italics).

Suppl. Fig. 2: Comparison of short-term variations in hydroclimate between low- and mid-latitudes in the NH. The residuals of the smoothed YOK-I and SU-96-2 records are shown in the middle of the figure, whereas long-term trends are shown at the bottom.

Suppl. Fig. 3: Crossplot between the residuals of the detrended time series of YOK-I δ^13^C^9^ and SU-96-2 band width^23^, presented in figure 4. The correlation coefficient between the two time series is r=0.51.

Supplementary References:

1. Tan, L. *et al.* Centennial- to decadal-scale monsoon precipitation variability in the semi-humid region, northern China during the last 1860 years: Records from stalagmites in Huangye Cave. *The Holocene* **21,** 287–296 (2011).

2. Zhang, P. *et al.* A Test of Climate, Sun, and Culture Relationships from an 1810-Year Chinese Cave Record. *Science* **322,** 940–942 (2008).

3. Tan, L. *et al.* A Chinese cave links climate change, social impacts, and human adaptation over the last 500 years. *Sci. Rep.* **5,** (2015).

4. Sinha, A. *et al.* Trends and oscillations in the Indian summer monsoon rainfall over the last two millennia. *Nat. Commun.* **6,** (2015).

5. Cosford, J., Qing, H., Mattey, D., Eglington, B. & Zhang, M. Climatic and local effects on stalagmite d13C values at Lianhua Cave, China. *Palaeogeogr. Palaeoclimatol. Palaeoecol.* **280,** 235–244 (2009).

6. Fensterer, C. *et al.* Cuban stalagmite suggests relationship between Caribbean precipitation and the Atlantic Multidecadal Oscillation during the past 1.3 ka. *The Holocene* (2012). doi:10.1177/0959683612449759

7. Sinha, A. *et al.* A 900-year (600 to 1500 A.D.) record of the Indian summer monsoon precipitation from the core monsoon zone of India. *Geophys. Res. Lett.* **34,** (2007).

8. Fleitmann, D., Burns, S. J., Neff, U., Mangini, A. & Matter, A. Changing moisture sources over the last 330,000 years in Northern Oman from fluid-inclusion evidence in speleothems. *Quat. Res.* **60,** 223–232 (2003).

9. Kennett, D. J. *et al.* Development and disintegration of Maya political systems in response to climate change. *Science* **338,** 788–791 (2012).

10. Buckley, B. M. *et al.* Climate as a contributing factor in the demise of Angkor , Cambodia. *Proc. Natl. Acad. Sci.* **107,** 6748–6752 (2010).

11. Haug, G. H., Hughen, K. A., Sigman, D. M., Peterson, L. C. & Röhl, U. Southward migration of the intertropical convergence zone through the Holocene. *Science* **293,** 1304–1308 (2001).

12. Shanahan, T. M. *et al.* Atlantic Forcing of Persistent Drought in West Africa. *Science* **324,** 377–380 (2009).

13. Reuter, J. *et al.* A new perspective on the hydroclimate variability in northern South America during the Little Ice Age. *Geophys. Res. Lett.* **36,** L21706 (2009).

14. Maupin, C. R. *et al.* Persistent decadal-scale rainfall variability in the tropical South Pacific Convergence Zone through the past six centuries. *Clim. Past* **10,** 1319–1332 (2014).

15. Bird, B. W., Abbott, M. B., Rodbell, D. T. & Vuille, M. Holocene tropical South American hydroclimate revealed from a decadally resolved lake sediment δ18O record. *Earth Planet. Sci. Lett.* **310,** 192–202 (2011).

16. Kanner, L. C., Burns, S. J., Cheng, H., Edwards, R. L. & Vuille, M. High-resolution variability of the South American summer monsoon over the last seven millennia : insights from a speleothem record from the central Peruvian Andes. *Quat. Sci. Rev.* **75,** 1–10 (2013).

17. Thompson, L. G. *et al.* Annually resolved ice core records of tropical climate variability over the past ~1800 years. *Science* **340,** 945–50 (2013).

18. Novello, V. F. *et al.* Centennial-scale solar forcing of the South American Monsoon System recorded in stalagmites. *Sci. Rep.* **6,** (2016).

19. Denniston, R. F. *et al.* Expansion and Contraction of the Indo-Pacific Tropical Rain Belt over the Last Three Millennia. *Sci. Rep.* **6,** (2016).

20. Haig, J., Nott, J. & Reichart, G.-J. Australian tropical cyclone activity lower than at any time over the past 550-1,500 years. *Nature* **505,** 667–71 (2014).

21. Sletten, H. *et al.* A petrographic and geochemical record of climate change over the last 4600 years from a northern Namibia stalagmite, with evidence of abruptly wetter climate at the beginning of southern Africa’s Iron Age. *Palaeogeogr. Palaeoclimatol. Palaeoecol.* **376,** 149–162 (2013).

22. Voarintsoa, N. R. G. *et al.* Stalagmite multi-proxy evidence of wet and dry intervals in northeastern Namibia : Linkage to latitudinal shifts of the Inter-Tropical Convergence Zone and changing solar activity from AD 1400 to 1950. *The Holocene* (2016). doi:10.1177/0959683616660170

23. Proctor, C. J., Baker, A., Barnes, W. L. & Gilmour, M. A. A thousand year speleothem proxy record of North Atlantic climate from Scotland. *Clim. Dyn.* **16,** 815–820 (2000).
